# The effectiveness of intra-aortic balloon pump for myocardial infarction in patients with or without cardiogenic shock: a meta-analysis and systematic review

**DOI:** 10.1186/s12872-016-0323-2

**Published:** 2016-07-08

**Authors:** Xiao-yun Zheng, Yi Wang, Yi Chen, Xi Wang, Lei Chen, Jun Li, Zhi-gang Zheng

**Affiliations:** Department of Senior Official Ward, China-Japan Friendship Hospital, 2 Yinghua Dongjie, Beijing, 100029 China

**Keywords:** Myocardial infarction, Percutaneous coronary intervention, Intra-aortic balloon pump

## Abstract

**Background:**

Conflicting reports on the efficacy of intra-aortic balloon pump (IABP) during percutaneous coronary intervention (PCI) incited us to evaluate the utility of IABP in patients with acute myocardial infarction (AMI).

**Methods:**

Randomized clinical trials comparing patients, who received IABP vs. control (no IABP) during PCI, were hand-searched from MEDLINE, Cochrane, and EMBASE databases using the terms “intra-aortic balloon pump, percutaneous coronary intervention, myocardial infarction, acute coronary syndrome”. Mortality rate (30-day and 6-month mortality) was the primary outcome, while the secondary outcomes included 30-day bleeding rate, reinfarction rate, revascularization rate and stroke rate.

**Results:**

Pooled results of the seven trials identified indicated that the 30-day and 6-month mortality rate were not significantly different between the IABP and control groups. However, in patients with MI, but without cardiogenic shock (CS), IABP was associated with lower odds of 30-day mortality (OR = 0.35, *p* = 0.015) and 6-month mortality (OR = 0.41, *p* = 0.020). The pooled results of 30-day bleeding rate was not significantly higher in patients with IABP than the control group, but for the patients with high risk PCI without CS, it was higher in patients with IABP than the control group (OR = 1.58, *p* = 0.009). The re-infarction, revascularization, and the stroke rate at 30 days of follow-up were not significantly different between the two groups.

**Conclusions:**

The present results do not favor the clinical utility of IABP in patients suffering high-risk PCI without CS and AMI complicated with CS. However, in patients with AMI, but without CS, IABP may reduce the 30-day and 6-month mortality rate.

## Background

Acute myocardial infarction (AMI) complicated by cardiogenic shock (CS) is one of the leading causes of death in patients hospitalized with AMI, and it accounts for 41.1 % of overall in-hospital mortality in a population-based study [1, 2]. Intra-aortic balloon pump (IABP) is the most widely used mechanical device for the treatment of AMI [3, 4], since its introduction by Kantrowitz and colleagues in early 1960s [5]. The International Benchmark Registry (250 US and non-US centers) of 22,633 AMI patients treated with IABP suggested that 19 % of IABP implantation were for cardiogenic shock, 19.9 % for angiography and angioplasty, and 14.6 % as an adjunct (pre-operative) to high-risk coronary artery bypass grafting [6]. IABP support effectively reduces the left ventricular wall stress and myocardial demand, increases the coronary perfusion pressure, stroke volume, cardiac output, and ameliorates ischemia, making it a potentially valuable therapy in CS [3, 7, 8]. Reports elsewhere suggest that IABP offers a substantial advantage when used in combination with thrombolytic therapy [9, 10]. In a previous study, the use of IABP in conjunction with thrombolytic therapy decreased the odds of death by 18 % [9]. In addition, IABP has been widely used in the prevention of adverse catheter laboratory events during elective high-risk PCI [11].

Despite its frequent use in the clinical practice for the treatment of AMI, recent reports dispute whether intra-aortic balloon counterpulsation provide any incremental benefit to reperfusion therapy [9, 10, 12, 13]. In patients with AMI and CS, the evidence in favor of IABP is currently limited to registry data and retrospective analyses, and small, prospective studies without any reliable mortality data [14]. A recent systematic review and meta-analysis comparing IABP versus no IABP in patients with AMI and CS concluded that the available data did not provide a convincing evidence for either benefit or harm to support the use of IABP counterpulsation [8]. According to the 2011 guidelines released by the American College of Cardiology Foundation (ACCF) and American Heart Association (AHA), IABP counterpulsation is reasonable in non-ST-elevation myocardial infarction (NSTEMI) patients for severe ischemia that is continuing or recurs frequently despite medical therapy, for hemodynamic instability in patients before or after coronary angiography, and for mechanical complications of MI [15]. In 2013, ACCF/AHA has released an updated guideline for patients with STEMI, where the recommendation for the placement of IABP in CS was downgraded from Class I to Class IIa, because of the lack of clear superiority in clinical benefit and reduction of mortality [16, 17]. Similarly, IABP was recommended in ST-elevation myocardial infarction (STEMI) patients with CS by the European Society of Cardiology in 2008 but an updated guideline released by European Society of Cardiology (ESC) and the European Association for Cardio-Thoracic Surgery (EACTS) in 2014 did not recommend routinely using IABP in patients with CS [18, 19].

Use of elective IABP support in patients undergoing high-risk PCI is still debatable. The evidence suggests that routine IABP use does not provide clinical benefit in patients undergoing high-risk procedures or those with AMI in the absence of CS, but it causes a relative reduction in the long-term all-cause mortality [11]. Bahekar and colleagues also indicated that while IABP is not beneficial in high-risk AMI patients without cardiogenic shock, there was significant reduction in mortality with IABP in patients having AMI with cardiogenic shock [20].

Considering the contrasting reports in the field and the necessity for unified guidelines for the use of IABP, it is of utmost importance to evaluate the clinical relevance of IABP as an adjunct therapy to PCI in patients with acute myocardial infarction with or without CS. The present meta-analysis evaluated the clinical outcomes of IABP as an adjunct therapy during PCI as opposed to PCI alone. The outcomes examined include, 30-day mortality, 6-month mortality, 30-day reinfarction rate, 30-day revascularization rate, 30-day stroke rate, and 30-day bleeding rate.

## Methods

### Search strategy

The current meta-analysis was conducted in accordance with the PRISMA guidelines [21]. We performed a literature search of the Pubmed, Medline, Cochrane, and EMBASE databases until December 31, 2015 using the key words, “intra-aortic balloon pump, percutaneous coronary intervention, myocardial infarction, acute coronary syndrome, and unstable angina”. The reference lists of relevant studies were also hand-searched.

### Selection criteria

Only randomized clinical trials in adult patient populations (≧18 years), who received PCI were included in the present meta-analysis. The intervention group received IABP during PCI, while the control group did not.

We excluded studies that are not-randomized controlled trials and those with no reported quantitative primary or secondary outcomes. Non-English articles, and non-original articles, including letters, comments, editorials, case reports, technical reports, and personal communications were also excluded from the analysis.

### Study selection and data extraction

Studies were identified by the search strategy by two independent reviewers. Where there was uncertainty regarding eligibility, a third reviewer was consulted.

Data extraction was also performed by two independent reviewers, and a third reviewer was consulted to resolve any discord. The following information was extracted from studies that met the inclusion criteria: the name of the first author, year of publication, study design, demographic data of subjects, patient diagnosis, prior medical history, type of intervention, and numerical data on outcomes of interest. The primary outcome analyzed was mortality rate (30-day mortality, 6-month mortality) while the secondary outcomes included 30-day bleeding rate, 30-day re-infarction rate, 30-day revascularization rate and 30-day stroke rate.

### Assessment of risk of bias

We utilized using the Cochrane Risk of Bias Tool to assess the included studies [22]. The quality assessment was performed by two independent reviewers and a third reviewer was consulted for any uncertainties.

### Statistical analysis

For each outcome analyzed, the odds ratio (OR) with 95 % confidence interval (CI) was calculated. Heterogeneity among the studies was assessed by the Cochran’s Q test and the I^2^ statistic. For Cochran’s Q, *p* < 0.10 indicated statistically significant heterogeneity. For the I^2^ statistic, which indicates the percentage of the observed between-study variability due to heterogeneity rather than chance, heterogeneity was categorized as follows: no heterogeneity, I^2^ = 0–25 %; moderate heterogeneity, I^2^ = 25–50 %; large heterogeneity, I^2^ = 50–75 %; and extreme heterogeneity, I^2^ = 75–100 %. All analyses were stratified according to subgroups based on patients’ risk factor profile (i.e., high-risk PCI without CS, MI with CS, and MI without CS). First, pooled ORs for each outcome within each group were calculated by the random-effects model of analysis (DerSimonian–Laird method) by assuming commonality in between-study variance across subgroups. Then, a random-effect model was performed to combine the estimates across subgroups. A two-sided *p* value < 0.05 was considered to indicate statistical significance. Sensitivity analysis was performed for the primary outcome, short-term mortality rate, based on the leave-one-out approach. Publication bias was assessed by constructing a funnel plot for primary outcome and using the Egger’s test. The absence of publication bias is indicated by the data points forming a symmetrical, funnel-shaped distribution and *p* > 0.05, as determined by the Egger’s test. All statistical analyses were performed using the statistical software, Comprehensive Meta-Analysis, version 2.0 (Biostat, Englewood, NJ, USA).

## Results

### Literature search

**Fig. 1:**
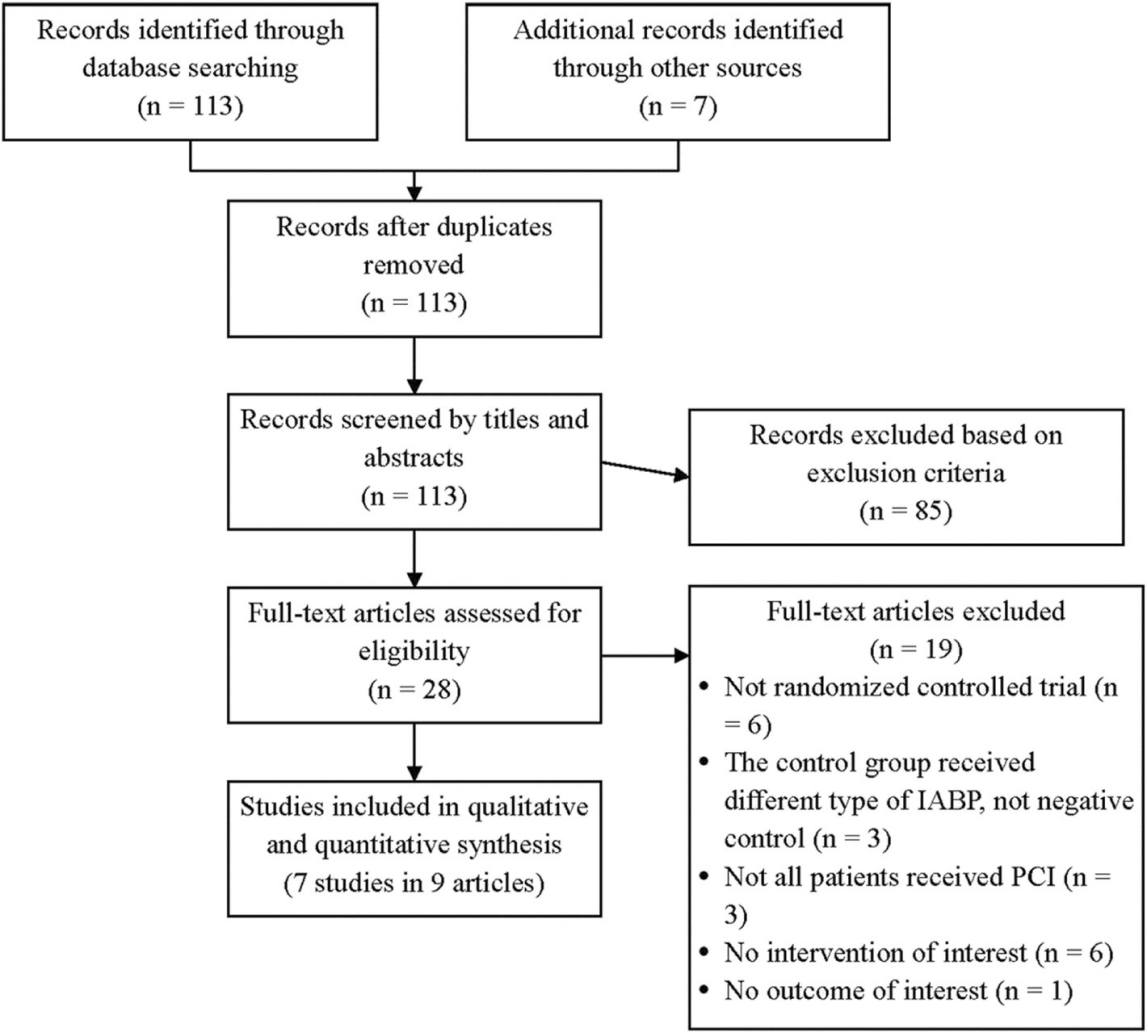
Flowchart of study selection. IABP, intra-aortic balloon pump; PCI: percutaneous coronary intervention

The flowchart for the selection of trials is outlined in Fig. 1. Of the 113 articles identified through the literature search, 87 were excluded, and the remaining 26 articles were assessed for full text reviewing. After full text reviewing, we excluded 17 articles for various reasons, including having no outcome of interest, the details of which are represented in Fig. 1. The present meta-analysis comprises seven randomized controlled trials reported in nine articles, which were included for the following qualitative and quantitative analysis [12, 13, 23–29].

### Study characteristics and outcomes

A total of seven randomized controlled trials were included in the meta-analysis. The characteristics of these studies are summarized in Table 1. Across the studies, the total numbers of patients ranged from 19 to 301 in the IABP group, while it ranged from 21 to 299 in the control (without IABP) group. Two studies (in three articles) were designed for patients with AMI complicated by CS [12, 13, 28], while two studies included patients with acute ST-elevation or non-ST-elevation myocardial infarction without CS [23, 24]. The remaining three studies (in four articles) were designed for high risk patients with MI without CS [25–27, 29].

**Table 1:** Summary of baseline characteristics of included studies in meta-analysis

Authors (Year)	Trial name	Comparison	Number of patients	Age (years)	Male (%)	Clinical symptom	Hypertension	Prior MI	Prior PCI	Prior CABG	Prior stroke
Thiele (2012, 2013) [12, 13]	IABP-SHOCK II	IABP	301	70 (58, 78)^a^	67.1	MI complicated by CS	213/296 (72.0 %)	71/300 (23.7 %)	63/299 (21.1 %)	20/300 (6.7 %)	24/300 (8.0 %)
		without IABP	299	69 (58, 76)^a^	70.6		199/299 (66.6 %)	61/299 (20.4 %)	52/299 (17.4)%	12/299 (4.0 %)	20/299 (6.7 %)
Gu (2011) [24]	--	IABP	51	67.4 (9.6)	56.9	Acute STEMI or non-STEMI without CS	35 (68.6 %)	2 (3.9 %)	NR	NR	NR
		without IABP	55	66.6 (8.0)	65.5		33 (60.0 %)	3 (5.5 %)	NR	NR	NR
Patel (2011) [23]	CRISP AMI	IABP	161	56.1 (48.3, 64.3)^a^	82.0	Acute STEMI without CS	39 (24.2 %)	NR	3 (1.9 %)	NR	0
		without IABP	176	57.7 (48.6, 66.4)^a^	81.8		60 (34.1 %)	NR	2 (1.1 %)	NR	1 (0.6 %)
Perera (2010, 2013) [26, 27]	BCIS-1	IABP	151	71 (9)	81	High risk PCI without CS	95 (63 %)	113 (75 %)	17 (11 %)	25 (17 %)	12 (8 %)
		without IABP	150	71 (10)	78		91 (61)	108 (73 %)	14 (9 %)	20 (13 %)	11 (7 %)
Prondzinsky (2010) [28]	IABP SHOCK	IABP	19	62.1 (38, 82)^b^	74	Acute MI complicated by CS	8 (42.1 %)	4 (21.1 %)	NR	NR	NR
		without IABP	21	66.1 (49, 82)^b^	81		10 (47.6 %)	5 (23.8 %)	NR	NR	NR
van't Hof (1999) [29]	--	IABP	118	59 (10)	84	High risk PCI without CS	NR	17 (14 %)	NR	3 (3 %)	NR
		without IABP	120	56 (11)	84		NR	16 (13 %)	NR	7 (6 %)	NR
Stone (1997) [25]	PAMI-II TRIAL	IABP	211	64.7 (11.9)	74.9	High risk MI without CS	116 (54.8 %)	45 (21.4 %)	NR	16 (7.5 %)	NR
		without IABP	226	63.7 (13.0)	75.2		126 (55.7 %)	49 (21.7 %)	NR	13 (5.9 %)	NR

*Abbreviations*: *IABP* intra-aortic balloon pump, *CS* cardiogenic shock, *MI* myocardial infarction, *STEMI* ST-elevation MI, *PCI* percutaneous coronary intervention, *CABG* coronary artery bypass graft, *NR* no reported

Data presented in mean (SD), median (IQR)^a^, or mean (range)^b^

Outcomes of the trials are shown in Table 2. The 30-day mortality rate ranged from 1.9 to 39.7 % in the IABP group, whereas, it ranged from 0.7 to 41.3 % in the control group. Similarly, the IABP group had a 6-month mortality rate of 1.9 to 48.7 %, while it was 5.2 to 49.2 % in the no IABP group. Patients in the IABP group demonstrated a 30-day re-infarction rate of 2 to 12.6 %, a 30-day revascularization rate of 0.7 to 20 %, a 30-day stroke rate of 0.7 to 2.4 %, and a 30-day bleeding rate of 3.1 to 36.0 %. Whereas, the control, no IABP group showed a 30-day re-infarction rate of 1.3 to 13.3 %, a 30-day revascularization rate of 1.8 to 22 %, a 30-day stroke rate of 0 to 1.7 %, and a 30-day bleeding rate of 1.7 to 27.4 % (Table 2).

**Table 2:** Summary of outcomes of included studies in meta-analysis

Authors (Year)	Comparison	Number of patients	30-day mortality	6-month mortality	30-day reinfarction rate	30-day revascularization rate	30-day stroke rate	30-day bleeding rate
Thiele (2012, 2013) [12, 13]	IABP	301	39.7 %	48.7 %	3.0 %	20 %	0.7 %	20.7 %
	without IABP	299	41.3 %	49.2 %	1.3 %	22 %	1.7 %	20.8 %
Gu (2011) [24]	IABP	51	9.8 %	17.6 %	2.0 %	3.9 %	NR	11.8 %
	without IABP	55	27.3 %	32.7 %	3.6 %	1.8 %	NR	3.6 %
Patel (2011) [23]	IABP	161	1.9 %	1.9 %	NR	NR	1.9 %	3.1 %
	without IABP	176	4.0 %	5.2 %	NR	NR	0.6 %	1.7 %
Perera (2010, 2013) [26, 27]	IABP	151	2.0 %	4.6 %	12.6 %	0.7 %	1.3 %	19.2 %
	without IABP	150	0.7 %	7.4 %	13.3 %	2.7 %	0	11.3 %
Prondzinsky (2010) [28]	IABP	19	36.8 %	NR	NR	NR	NR	NR
	without IABP	21	28.6 %	NR	NR	NR	NR	NR
van't Hof (1999) [29]	IABP	118	NR	10 %	NR	NR	NR	NR
	without IABP	120	NR	8 %	NR	NR	NR	NR
Stone (1997) [25]	IABP	211	4.3 %	NR	6.2 %	4.7 %	2.4 %	36.0 %
	without IABP	226	3.1 %	NR	8.0 %	4.0 %	0	27.4 %

*Abbreviations*: *IABP* intra-aortic balloon pump, *NR* no reported

### Meta-analysis

#### Primary outcome: 30-day mortality rate and 6-month mortality rate

**Fig. 2:**
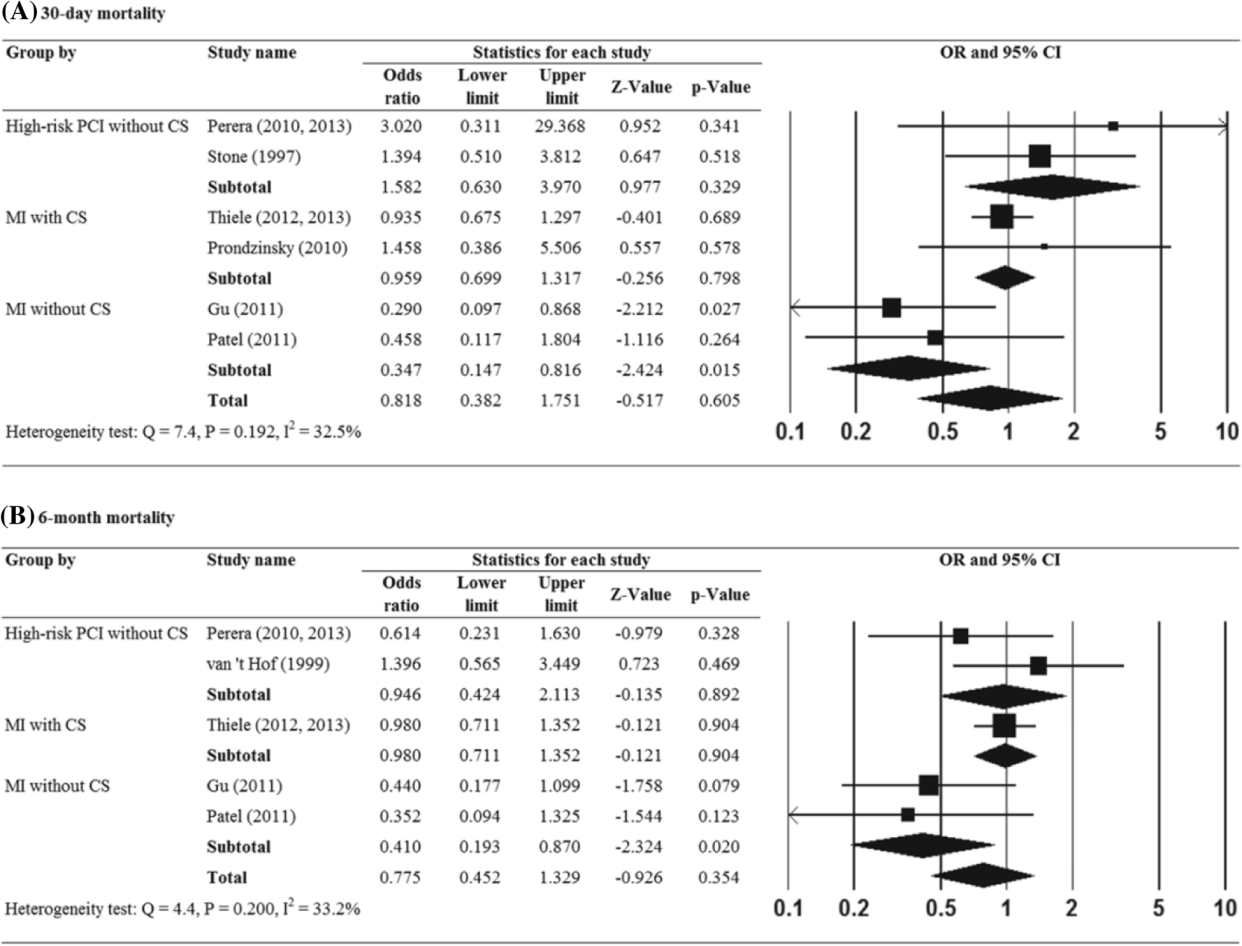
Forest plots showing the results for the meta-analysis of (**a**) 30-day mortality rate, (**b**) 6-month mortality rate. Abbreviations: IABP, intra-aortic balloon pump; CI, confidence interval

One study [29] was excluded from the analysis, because it had not reported a 30-day mortality rate. There was no evidence of significant heterogeneity when data from the remaining six studies were pooled (Q = 7.4, *p* = 0.192; I^2^ = 32.5 %). The overall analysis revealed that the 30-day mortality rate was not significantly different in patients with IABP, as compared to patients without IABP (OR = 0.82, 95 % CI = 0.38 to 1.75, Z = −0.52, *p* = 0.605) (Fig. 2a). In patients with MI, but without CS, the likelihood of 30-day mortality was significantly lower in patients with IABP than in those without IABP (OR = 0.35, 95 % CI = 0.15 to 0.82, Z = −2.42, *p* = 0.015) (Fig. 2a).

Two studies [25, 28] were excluded from the final analysis as they did not report the 6-month mortality rate. There was no evidence of significant heterogeneity when data from the five studies were pooled (Q = 4.4, *p* = 0.200; I^2^ = 33.2 %) (Fig. 2b). The overall analysis revealed that the 6-month mortality rate was not significantly different in patients with IABP, as opposed to those without IABP (OR = 0.78, 95 % CI = 0.45 to 1.33, Z = −0.93, *p* = 0.354). Similarly, in patients with MI, but without CS, IABP was associated with lower odds of 6-month mortality (OR = 0.41, 95 % CI = 0.19 to 0.87, Z = −2.324, *p* = 0.020) (Fig. 2b).

#### Secondary outcomes

##### 30-day bleeding rate

Two studies [28, 29] were excluded from the analysis as there was no report on the 30-day bleeding rate. There was no evidence of significant heterogeneity when data from the five studies were pooled (Q = 5.0, *p* = 0.288; I2 = 19.9 %) (Table 3). The overall analysis revealed that the 30-day bleeding rate was significantly higher in patients with IABP compared to those without IABP (OR = 1.39, 95 % CI = 0.86 to 2.24, Z = 1.36, *p* = 0.174). The results of subgroup analysis showed that patients with IABP had higher odds of 30-day bleeding than those without IABP in the subgroup of high-risk PCI without CS (OR = 1.59, 95 % CI = 1.12 to 2.24, Z = 2.63, *p* = 0.009) (Table 3).

**Table 3:** Results of meta-analysis for secondary outcomes

Outcomes	No. of studies	OR (95 % CI)	*P*
30-day bleeding			
High-risk PCI without CS	2	1.585 (1.124, 2.235)	0.009*
MI with CS	1	0.992 (0.668, 1.472)	0.967
MI without CS	2	2.451 (0.826, 7.273)	0.106
Total	5	1.391 (0.864, 2.238)	0.174
30-day reinfarction rate			
High-risk PCI without CS	2	0.851 (0.517, 1.400)	0.525
MI with CS	1	2.273 (0.692, 7.464)	0.176
MI without CS	1	0.530 (0.047, 6.028)	0.609
Total	4	0.964 (0.614, 1.514)	0.875
30-day revascularization rate			
High-risk PCI without CS	2	0.749 (0.180, 3.118)	0.691
MI with CS	1	0.883 (0.501, 1.554)	0.665
MI without CS	1	2.204 (0.194, 25.071)	0.524
Total	4	0.900 (0.538, 1.505)	0.688
30-day stroke rate			
High-risk PCI without CS	2	7.959 (0.974, 65.021)	0.053
MI with CS	1	0.393 (0.076, 2.043)	0.267
MI without CS	1	3.323 (0.342, 32.271)	0.301
Total	4	1.576 (0.511, 4.861)	

*Abbreviations*: *CS* cardiogenic shock, *MI* myocardial infarction

* *P* < 0.05

##### 30-day reinfarction rate

Three studies [23, 28, 29] were excluded from the analysis, since a 30-day re-infarction rate was not reported. There was no evidence of significant heterogeneity when data from the remaining four studies were pooled (Q = 2.64, *p* = 0.450; I^2^ = 0 %). The overall analysis revealed that the 30-day reinfarction rate was not significantly different in patients with IABP as compared to patients without IABP (OR = 0.96, 95 % CI = 0.61 to 1.51, Z = −0.16, *p* = 0.875). No significant results were observed in the subgroup analysis according to the patients’ risk factor profile (Table 3).

##### 30-day revascularization rate

Three studies [23, 28, 29] were excluded from the analysis as the 30-day revascularization rate was not reported. There was no evidence of significant heterogeneity when data from the four studies were pooled (Q = 2.23, *p* = 0.526; I^2^ = 0 %). The overall analysis revealed that the 30-day revascularization rate was not significantly different between the two groups (IABP vs. control, no IABP) (OR = 0.90, 95 % CI = 0.54 to 1.51, Z = −0.40, *p* = 0.688). No significant results were found in the subgroup analysis based on patients’ risk factor profile (Table 3).

##### 30-day stroke rate

Three studies [24, 28, 29] were excluded from the final analysis as no data on 30-day stroke rate was reported. There was no evidence of significant heterogeneity when data from the remaining four studies were pooled (Q = 5.59, *p* = 0.133; I^2^ = 46.3 %). The overall analysis revealed that the 30-day stroke rate was not significantly different in patients with IABP as compared to those without IABP (OR = 1.58, 95 % CI = 0.51 to 4.86, Z = 0.79, *p* = 0.428). No significant results were found in the subgroup analysis according to the patient risk factor profile (Table 3).

### Sensitivity analysis

**Fig. 3:**
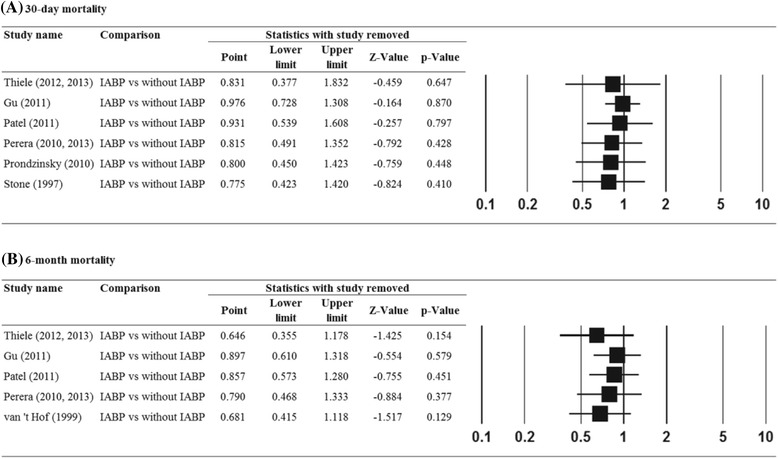
Results of sensitivity analysis to examine the influence of individual studies on pooled estimates as determined using the leave-one-out approach: (**a**) 30-day mortality rate, (**b**) 6-month mortality rate. Abbreviations: IABP, intra-aortic balloon pump; CI, confidence interval

The results of the meta-analysis using the leave-one-out approach to assess sensitivity is summarized in Fig. 3a (30-day mortality) and Fig. 3b (6-month mortality). For 30-day bleeding rate, 30-day reinfarction rate, 30-day revascularization rate, and 30-day stroke rate, results were not shown. The direction and magnitude of the pooled estimates for 30-day mortality and 6-month mortality did not vary considerably, indicating that the meta-analysis had good reliability.

### Publication bias

**Fig. 4:**
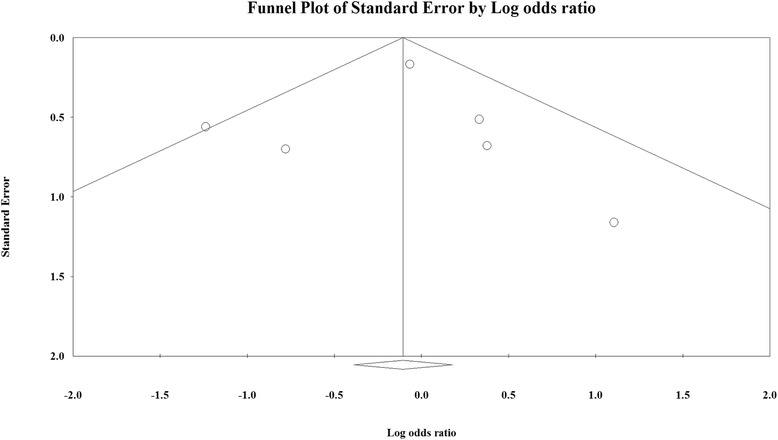
Funnel plot for publication bias for 30-day mortality. White circles represent observed studies. White rhombuses represent observed combined effect size

There was no evidence of significant publication bias for the 30-day mortality rate as assessed by the Egger’s test (Fig. 4). The results reveal no publication bias for the 30-day mortality rate (*p* = 0.472). Publication bias was not assessed for the other five outcomes, because more than five studies are required to detect funnel plot asymmetry [30].

### Assessment of risk of bias

**Fig. 5:**
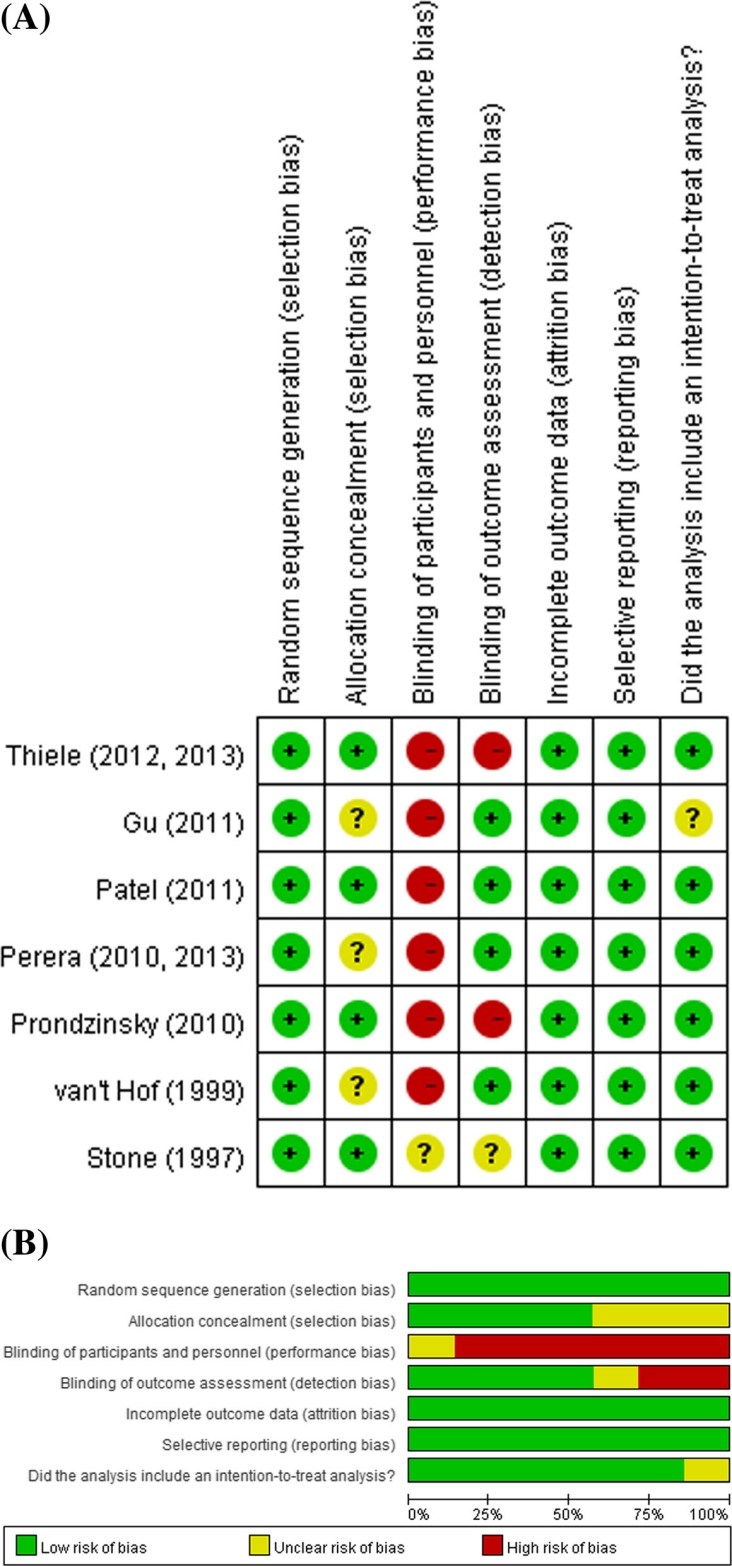
Summary of quality assessment. **a** Risk of potential bias of individual study, **b** Risk of bias of all included studies

The results of the assessments of risk of bias are presented in Fig. 5. The risk of potential bias of individual studies is given in Fig. 5a, while the risk of bias of all included studies is represented in Fig. 5b. The bias in the results can be mainly attributed to the performance bias, because none of the included studies could blind the patients or the study personnel in-charge.

## Discussion

IABP increase diastolic aortic pressure, which improves the diastolic coronary flow. IABP simultaneously reduces systolic aortic pressure, which in turn decrease the afterload and oxygen consumption of the myocardium [3, 8, 29]. In addition to AMI, IABP has been widely used in indications including, CS, high risk percutaneous coronary intervention and cardiac surgery for hemodynamic support [3, 14]. Besides its long-standing clinical use as the main form of mechanical circulatory support, the long-term benefits of IABP are still controversial due to the paucity of prospective, randomized clinical trials [14]. The current study was undertaken to broaden our understanding through a systematic review and meta-analysis of the existing literature in the clinical practice of IABP in terms of AMI with/without CS as well as in patients with high risk PCI. The pooled results of the current meta-analysis do not favor the overall survival and hence, the clinical utility of IABP, in patients suffering high-risk PCI without CS and AMI complicated with CS. However, for patients with MI but without CS, IABP may reduce 30-day and 6-month mortality rate.

CS is a clinical state of hypoperfusion characterized by a systolic pressure, 90 mmHg and a central filling pressure (wedge pressure), .20 mmHg, or a cardiac index,1.8 L/min/m2, and caused by the extensive loss of viable myocardial tissue. IABP is recommended by ACCF/AHA guideline (2013), which stated that “The use of IABP counterpulsation can be useful for patients with cardiogenic shock after STEMI who do not quickly stabilize with pharmacological therapy. (Class IIa recommendation, Level of evidence: A)” [16]. However, Sjauw et al. (2009) have challenged the existing general recommendations for the use of IABP in patients with ST-segment elevation myocardial infarction (STEMI) complicated by CS and have confirmed that IABP do not offer any advantage during PCI [10]. These findings were further supported by a well-powered, prospective, randomized clinical trial (IABP-SHOCK II trial) [12, 13], where they demonstrated that IABP did not reduce 30-day mortality or 12 month all-cause mortality in patients undergoing early revascularization for myocardial infarction complicated by cardiogenic shock. In addition, in patients with acute anterior STEMI without shock, no reduction in the infarct size was noted for IABP along with PCI as compared to PCI alone [23]. The current results are in agreement with the previous meta-analyses, [8, 10] where the benefit of adjunctive IABP therapy was not statistically significant in STEMI patients complicated by CS, and IABP did not show a significant reduction of mortality in patients with AMI and cardiogenic shock. In an updated ESC/EACTS Guidelines in 2014, routine use of IABP in patients with cardiogenic shock was not recommended (Class III recommendation, Level of evidence: A) [19]. IABP insertion should only be considered in patients with haemodynamic instability/cardiogenic shock due to mechanical complications (Class IIa recommendation, Level of evidence: C) [19].

The role of IABP support in the management of high-risk patients with AMI remains unclear. In a EUROTRANSFER registry of unselected patients with STEMI complicated by CS, the long term outcomes were similar between the high risk population with IABP and the low risk non-IABP patients, indicating that IABP may be effective in high risk patients [31]. Our result is consistent with the previous systematic review and meta-analysis that, in high-risk STEMI patients without CS, the majority of the studies could not demonstrate an efficacy benefit for IABP as compared to the control group in terms of in-hospital mortality, left ventricular ejection fraction, and rate of recurrent ischemia [20, 32].

However, in patients with MI without CS, our meta-analysis showed a different result from the previous reports. A recent meta-analysis reported by Ahmad et al. found that IABP treatment had no statistically significant effect on mortality. This outcome was consistent when the articles were stratified by the presence or absence of CS [33]. A meta-analysis of six randomized trials concluded that IABP did not reduce all-cause death [34]. In a systematic review including randomized controlled trials published between 1981 and 2011, the majority of the studies could not demonstrate a beneficial effect of IABP therapy in patients with STEMI without CS [32]. Moreover, a retrospective analysis on the role of IABP in patients with acute MI without CS also found no difference in the in-hospital rate of cardiac death among patients who received IABP at the time of their coronary revascularization and the control group [35]. However, we found that IABP may reduce 30-day and 6-month mortality rate in patients with MI, but without CS. This is in agreement with a systematic review reported by Ye et al. where they reported that while IABP did not reduce mortality within 2 months and 6–12 months of intervention in AMI patients with CS, but it can reduce 6–12 month mortality in patients with AMI without CS [36]. Since we only included two RCTs in this subgroup, the clinical benefit of IABP in patients with acute MI without CS remains to be explored further in future studies.

Though IABP did not offer any major advantage during primary PCI, it was effective as an adjunctive therapy to thrombolysis in patients with MI. Subgroup analysis even showed that, in patients with high-risk PCI without CS, IABP had higher odds of 30-day bleeding than those without IABP. Correspondingly, we did not observe any difference in the secondary outcomes like, 30-day mortality rate, 6-month mortality, 30-day reinfarction rate, 30-day revascularization rate, or 30-day stroke rate between the IABP and the control groups. The meta-analysis reported by Cassese et al. had similar conclusion that IABP significantly reduced recurrent myocardial ischemia and increased the risk of bleeding [34]. The meta-analysis reported by Bahekar et al. also found that IABP significantly increased the risk of moderate to major bleeding [20]. Major bleeding associated with IABP, thus requiring increased transfusion, was also demonstrated in retrospective studies [35]. For revascularization rate after treatment, the meta-analysis reported by Sjauw et al. found that IABP showed a significantly higher revascularization rate compared to patients without support [10].

Though the majority of the recent reviews had failed to demonstrate a survival benefit for IABP, it was shown to have some beneficial effect on hemodynamic parameters, like cardiac index, mean arterial pressure, and pulmonary capillary wedge pressure [8]. It has been suggested that the use of IABP should be reserved for patients with severe hemodynamic compromise [29]. However, it should be noted that the improved hemodynamic status does not always translate into improved survival outcomes. In a recent commentary, Grieshaber and colleagues have suggested that IABP might have a greater effect in patients with reduced coronary perfusion and those with severely reduced left ventricular function, like in patients with AMI who need to be temporized prior to cardiac bypass surgery [7]. We did not analyze the hemodynamic parameters due to the limited number of data available in our included studies.

The current meta-analysis is an updated review of the available data on the utility of IABP during PCI. However, there are several limitations to our analysis, including the limited number of included studies in each subgroup and the heterogeneity in patient characteristics among the studies. The publication bias is difficult to interpret due to the limited number of included studies. Furthermore, there is a potential bias resulting from the inadequate blinding of patients and the study personnel.

## Conclusions

The included RCTs demonstrate that IABP may reduce 30-day and 6-month mortality rate in patients with MI but without CS. In addition, for the patients with high risk PCI without CS, receiving IABP may have higher 30-day bleeding rate in compared to those without IABP. No clinical benefit of IABP was demonstrated in patients suffering high-risk PCI without CS and AMI complicated with CS. Future studies comprising of large, multicentric, prospective randomized trials should be undertaken to validate the current data and also to confirm or disprove its efficacy in patients with different diagnosis, including patients with high to moderate risk of CS and patients with AMI with and without CS.

## Abbreviations

AMI, acute myocardial infarction; CABG, coronary artery bypass graft; CS, cardiogenic shock; IABP, intra-aortic balloon pump; MI, myocardial infarction; PCI, percutaneous coronary intervention; STEMI, ST-segment elevation myocardial infarction
